# Targeting AMP-activated protein kinase in adipocytes to modulate obesity-related adipokine production associated with insulin resistance and breast cancer cell proliferation

**DOI:** 10.1186/1758-5996-3-16

**Published:** 2011-07-20

**Authors:** Jean Grisouard, Kaethi Dembinski, Doris Mayer, Ulrich Keller, Beat Müller, Mirjam Christ-Crain

**Affiliations:** 1 Department of Biomedicine, University Hospital Basel, Basel, CH-4031, Switzerland; 2 Hormones and Signal Transduction, German Cancer Research Centre, DKFZ-ZMBH Alliance, Heidelberg, D-69120, Germany; 3 Division of Endocrinology, Diabetes and Clinical Nutrition, University Hospital Basel, Basel, CH-4031, Switzerland; 4 Medical University Clinic, Kantonsspital Aarau, Aarau, CH-5001, Switzerland

## Abstract

**Background:**

Adipokines, e.g. TNFα, IL-6 and leptin increase insulin resistance, and consequent hyperinsulinaemia influences breast cancer progression. Beside its mitogenic effects, insulin may influence adipokine production from adipocyte stromal cells and paracrine enhancement of breast cancer cell growth. In contrast, adiponectin, another adipokine is protective against breast cancer cell proliferation and insulin resistance.

AMP-activated protein kinase (AMPK) activity has been found decreased in visceral adipose tissue of insulin-resistant patients. Lipopolysaccharides (LPS) link systemic inflammation to high fat diet-induced insulin resistance. Modulation of LPS-induced adipokine production by metformin and AMPK activation might represent an alternative way to treat both, insulin resistance and breast cancer.

**Methods:**

Human preadipocytes obtained from surgical biopsies were expanded and differentiated *in vitro *into adipocytes, and incubated with siRNA targeting AMPKalpha1 (72 h), LPS (24 h, 100 μg/ml) and/or metformin (24 h, 1 mM) followed by mRNA extraction and analyses. Additionally, the supernatant of preadipocytes or derived-adipocytes in culture for 24 h was used as conditioned media to evaluate MCF-7 breast cancer cell proliferation.

**Results:**

Conditioned media from preadipocyte-derived adipocytes, but not from undifferentiated preadipocytes, increased MCF-7 cell proliferation (p < 0.01). Induction of IL-6 mRNA by LPS was reduced by metformin (p < 0.01), while the LPS-induced mRNA expression of the naturally occurring anti-inflammatory cytokine interleukin 1 receptor antagonist was increased (p < 0.01). Silencing of AMPKalpha1 enhanced LPS-induced IL-6 and IL-8 mRNA expression (p < 0.05).

**Conclusions:**

Adipocyte-secreted factors enhance breast cancer cell proliferation, while AMPK and metformin improve the LPS-induced adipokine imbalance. Possibly, AMPK activation may provide a new way not only to improve the obesity-related adipokine profile and insulin resistance, but also to prevent obesity-related breast cancer development and progression.

## Introduction

Breast cancer is the most frequent cancer type among women worldwide, and 21% of all breast cancer deaths worldwide are estimated to be attributable to obesity and physical inactivity [1,2]. The incidence of type 2 diabetes mellitus (T2DM) is presumed to be a direct result of the obesity epidemic [3]. The breast cancer risk is increased in diabetic women independently from obesity [4,5]. Thus, both, obesity and diabetes are risk factors for breast cancer and the prevalence of each of these diseases will continue to rise worldwide [6].

Adipokines are polypeptides produced and secreted by the adipose tissue and dysregulation of their production and secretion in the obese state leads to obesity-related complications [7,8]. Invasive breast tumours break through the basement membrane and infiltrate fibrous tissue barriers, resulting in an immediate juxtaposition of adipocytes and breast cancer cells, thus allowing paracrine interactions between the two cell types [9]. Insulin resistance and consequent hyperinsulinaemia may be a common factor linking T2DM and cancer [10]. Beside its stimulating effects on breast cancer cell proliferation [11], insulin may influence adipokine production from adipose tissue and adipocyte stromal cells and may indirectly reinforce cancer cell growth. Similarly, insulin resistance-induced hyperglycaemia and hyperlipidaemia may alter adipokine production in adipocytes and enhance breast cancer development [12].

Chronic low grade systemic inflammation is associated with obesity and insulin resistance. Lipopolysaccharide (LPS) from the gut microbiota is a triggering factor linking inflammation to high fat diet-induced metabolic syndrome [13,14]. Metformin is the first line oral antidiabetic drug for patients with T2DM [15]. AMP-activated protein kinase (AMPK) targets cytokine secretion from the adipose tissue and the activation of this kinase by metformin could explain the beneficial effects of this drug on inflammation [16].

In view of these relationships, we aimed to assess the effect of low grade inflammation induced by LPS and of metformin on adipokine production in human adipocytes and to observe the stimulatory effect of adipocyte-secreted factors on breast cancer cell proliferation.

## Materials and methods

The study was approved by the local Ethics Committee and informed consent was obtained from patients. Subcutaneous fat tissue samples were obtained from obese donors (BMI > 30 kg/m^2^, males and females, mean age 47 years) during elective abdominal surgery performed for various non-malignant conditions.

Preadipocytes were isolated, expanded *in vitro *until confluence and subjected to adipogenic differentiation medium for 14 days as previously described [17,18]. After differentiation, adipocytes were washed twice with warm phosphate buffered saline (PBS) and were kept for 48 h in low glucose (5 mM) medium. Then, adipocytes were pre-incubated with 1 mM metformin (Sigma-Aldrich, Buchs, Switzerland) for 1 h followed by treatment with 100 ng/ml LPS (Sigma-Aldrich). AMPKα1 silencing was performed as previously described [18].

Low glucose DMEM medium with 2% dextran-coated charcoal-treated heat-inactivated FCS (DCC-FCS, Lubio Science, Luzern, Switzerland) was added to confluent preadipocytes or preadipocyte-derived adipocytes. After 24 h in culture, the supernatant or conditioned medium was collected, filtered with a 0.2 μm syringe filter and stored at -20°C.

MCF-7 breast cancer cells were maintained in DMEM (25 mM glucose) containing 10% FCS (Lubio Science). Then, cells were grown in medium supplemented with 10% DCC-FCS for 72 h. 1 × 10^4 ^cancer cells/well were plated in a 96-well plate in medium containing 2% DCC-FCS for 24 h and stimulated every 24 h for a total of 72 h with low glucose DMEM + 2% DCC-FCS or conditioned medium. At the end of incubation time, cells were washed twice with PBS, fixed for 5 min with 100 μl of 3% paraformaldehyde and stained for 10 min with 100 μl of 1% crystal violet dye dissolved in 10% ethanol. Plates were extensively washed with water to remove traces of unbound crystal violet dye. After air drying, the bound dye was dissolved in 100 μl of 10% acetic acid. Optical density was read at 595 nm using a plate reader (Bucher biotec, Basel, CH) [11].

For quantitative analysis of adipokine expression, RNA was isolated and 1 μg total RNA was subjected to reverse transcription-PCR. cDNA was subjected to quantitative real-time PCR analysis using the power Sybr^®^-Green PCR master mix (Applied Biosystems) and the ABI 7500 Sequence detection system [19]. Primers were designed as follows: IL-6 sense primer, 5'-TCTTCAGAACGAATTGACAAACAAA-3', IL-6 antisense primer, 5'-GCTGCTTTCACACATGTTACTCTTG-3', IL-8 sense primer, 5'-GCCATAAAGTCAAATTTAGCTGGAA-3', IL-8 antisense primer, 5'-GTGCTTCCACATGTCCTCACA-3'; interleukin 1 receptor antagonist (IL-1RA) sense primer, 5'-TGCCTGTCCTGTGTCAAGTC-3' and IL-1RA antisense primer, 5'-TCTCGCTCAGGTCAGTGATG-3'. Hypoxanthine-guanine phosphoribosyltransferase (HPRT) primers were used as loading control (HPRT sense primer, 5'-TCAGGCAGTATAATCCAAAGATGGT-3' and HPRT antisense primer, 5'-AGTCTGGCTTATATCCAACACTTC-3'.

Data are presented as mean ± standard deviation (SD) from a minimum of three independent experiments. One-way analysis of variance was performed and the Tukey's posthoc multiple comparison test was applied. Overall, a *P *value < 0.05 was considered significant.

## Results

**Figure 1:**
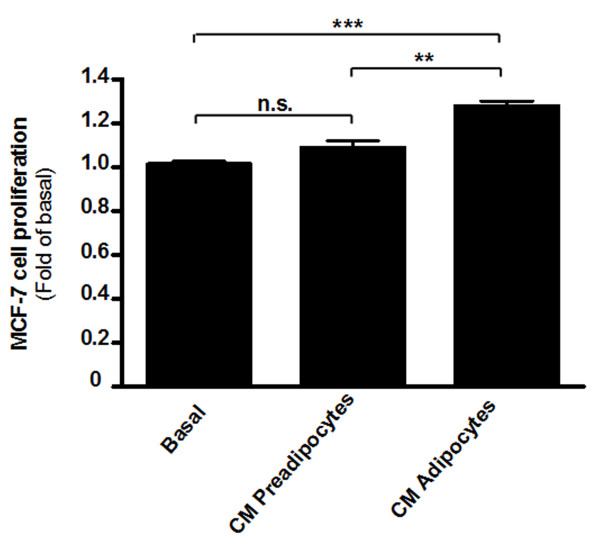
**Effect of adipocyte conditioned medium (CM) on breast cancer cell proliferation**. MCF-7 breast cancer cells were incubated for 72 h with low glucose DMEM medium (basal), low glucose DMEM conditioned medium from preadipocytes (CM Preadipocytes) or low glucose DMEM conditioned medium from preadipocyte-derived adipoytes (CM Adipocytes). Then, proliferation of MCF-7 cells was measured by colorimetric method using crystal violet dye. Results are expressed as fold of basal (**, p < 0.01 vs. CM Preadipocytes; ***, p < 0.001 vs. basal; n.s., not significant).

Conditioned medium (CM) from preadipocytes and preadipocyte-derived adipocytes (14 days after differentiation) was collected and breast cancer MCF-7 cell proliferation was evaluated after 72 h in these media (5 mM glucose) (Figure 1). DMEM medium (5 mM glucose) was used as basal. CM from preadipocytes did not increase MCF-7 cell proliferation (p = n.s.). However, CM from preadipocyte-derived adipocytes increased MCF-7 cell proliferation to 1.28 ± 0.02 fold (p < 0.001 vs. basal and p < 0.01 vs. CM preadipocytes).

**Figure 2:**
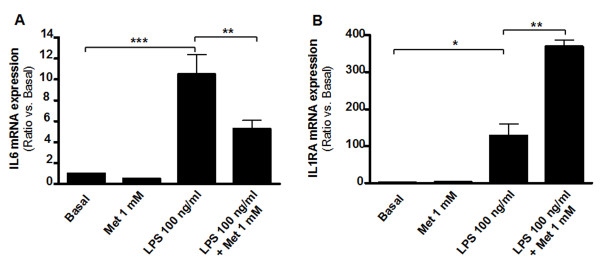
**Metformin modulates LPS-induced adipocytokine expression**. Human subcutaneous preadipocyte-derived adipocytes were pre-incubated for 1 h with 1 mM metformin followed by stimulation with 100 ng/ml LPS for 24 h. mRNA expression of IL-6 (**A**) and interleukin-1 receptor antagonist (IL1RA) (**B**) were analysed by quantitative real-time PCR. (*, p < 0.05 vs. basal; **, p < 0.01 vs. LPS; ***, p < 0.001 vs. basal; n.s., not significant).

Incubation of preadipocyte-derived adipocytes with 100 ng/ml LPS increased IL-6 mRNA expression to 10.5 ± 1.9 fold (p < 0.001 vs. basal)(Figure 2A) and induction of IL-6 mRNA by LPS was significantly reduced by 1 mM metformin to 5.3 ± 0.8 fold (p < 0.01 vs. LPS). Additionally, 1 mM metformin enhanced LPS-induced mRNA expression of IL-1RA, a naturally occurring anti-inflammatory cytokine (p < 0.01 vs. LPS) (Figure 2B).

**Figure 3:**
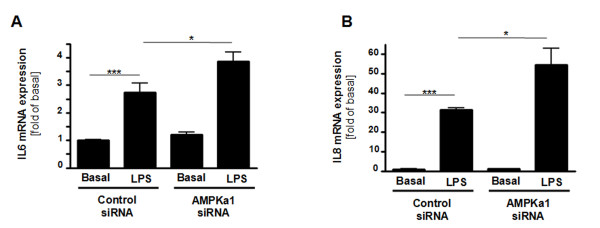
**AMPKalpha1 prevents the LPS-induced IL-6 and IL-8 adipocytokine expression**. Human subcutaneous preadipocyte-derived adipocytes were transfected for 72 h with control non-targeting siRNA pool (Control siRNA) or siRNA pool specifically targeting AMPKalpha1 (AMPKα1 siRNA) and incubated for 24 h with 100 ng/ml LPS. mRNA expression of IL-6 (**A**) and IL-8 (**B**) was analysed by quantitative real-time PCR. (*, p < 0.05 vs. LPS in control siRNA cells; ***, p < 0.001 vs. basal in control siRNA cells).

We silenced AMPKα1 subunit in preadipocyte-derived adipocytes to check its involvement in the effects of LPS on adipocytokine mRNA expression. Using siRNA pool specifically targeting AMPKα1, AMPKα1 mRNA expression and protein content were decreased to 0.28 ± 0.03 and 0.50 ± 0.08 fold, respectively (p < 0.001 vs. control siRNA)(data not shown). AMPKα1 silencing enhanced LPS-induced IL-6 and IL-8 mRNA expression (Figure 3, p < 0.05 vs. LPS in control siRNA cells).

## Discussion

**Figure 4:**
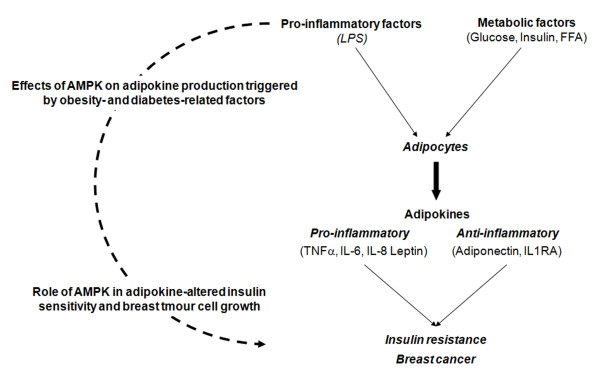
**Summary of our research aims and hypotheses**. Our preliminary results suggest that AMPK modulates the adipokine expression and secretion profiles from human adipocytes upon stimulation by inflammatory and/or metabolic factors. Imbalance in the adipokine profile triggers insulin resistance and breast cancer cell growth. Moreover, adipokines alter AMPK activity and modulation of this activity might inhibit the downstream effects of pro-inflammatory and pro-proliferative adipokines (TNFα, IL-6, IL-8, leptin) and improve the effects of anti-inflammatory and anti-proliferative adipokines (adiponectin, IL1-RA) in insulin resistant tissues and breast tumours. AMPK: AMP-dependent protein kinase, LPS: lipopolysaccharides, FFA: free fatty acids, TNFα: tumour necrosis factor α, IL-6: interleukin 6, IL-8: interleukin-8, IL1RA: interleukin-1 receptor antagonist).

Decreased AMPK activity has been found in visceral adipose tissue of patients with central obesity due to Cushing's syndrome [20] and of obese insulin-resistant individuals [21]. This suggests a central role for this enzyme in obesity and related insulin resistance. We therefore aimed to investigate how AMPK modulates adipokine production triggered by obesity- and type 2 diabetes mellitus (T2DM)-related factors and how such a modulation may prevent insulin resistance and breast tumour cell proliferation. In turn, adipokines alter AMPK activity and might play a crucial role in adipokine-altered insulin sensitivity and breast tumour cell growth (Figure 4). Our findings suggest that conditioned medium from human adipocytes increase breast cancer cell proliferation. Our data also show that metformin and AMPK alter LPS-induced adipokine expression, favoring the anti-inflammatory adipokine expression and decreasing pro-inflammatory adipokine expression in human adipocytes.

Interestingly, CM obtained from murine 3T3-L1 fully differentiated adipocytes induced growth of MCF-7 cells and such changes were attributed to reduced apoptosis rather than increased proliferation [22]. In our study, apoptosis was not analysed by specific assays. However, floating dead cells were removed by washing of the cell layer with PBS to eliminate most of the dead cells before fixation and staining. Nevertheless, we cannot exclude that apoptotic cells still attached to the culture dish were also stained by crystal violet under our experimental conditions. Further experiments will be needed to expand our findings in other parameters which are important for tumour progression such as apoptosis, invasion and migration.

Additionally, this doctoral thesis reported that conditioned media from murine preadipocytes was less efficient on MCF-7 cell proliferation and that low glucose medium reduced the effect of murine adipocyte conditioned media on MCF7 growth [22]. Since incubation with low glucose medium reduced IGF1mRNA levels in adipocytes and since adipocyte conditioned media proliferative effect was reverted by inhibiting the IGF-1 pathway, the author suggests that adipocyte-produced IGF1 has a crucial role in promoting cancer cell growth [22]. Similar results were obtained regarding human preadipocyte conditioned media in our study. To avoid the possible effects of high glucose concentration on adipocyte secretion such as IGF in the culture supernatant, our human adipocytes were incubated in low glucose (5 mM) medium before collecting the conditioned media. Low glucose medium might prevent the IGF release and this suggests that other adipokines are involved in breast cancer cell growth.

To obtain our data, both male and female adipose tissues were considered since the number of tissue available from females was not sufficient. The small size of the studied cohort of patients does not allow drawing conclusions about any gender specific differences in proliferation. Further work should be performed and adipose tissues from females are certainly more relevant regarding breast cancer proliferation. Ultimately, difference in a variety of parameters between fat depots is important and fat tissue isolated from the breast should be considered. Also, discrepancies between adipocytes from different donors were observed and the results were not consistent for both LPS-induced IL-1RA mRNA expression after AMPKα1-silencing and LPS-induced IL-8 mRNA expression upon metformin incubation (data not shown). This suggests that metformin and AMPK might alter the expression of some adipokines (e.g. IL-6) in a similar way and expression of some other adipokines in a different manner. Therefore, metformin might have some AMPK-dependent and -independent effects, which could be unraveled if AMPK expression is silenced upon metformin incubation. Further experiments also need to be performed to check the effects of metformin and AMPK modulation of adipokine expression on breast cancer cell proliferation.

Recent studies demonstrated that metformin treatment increased adiponectin plasma levels in women with polycystic ovary syndrome (PCOS) [23] and T2DM patients [24] while metformin treatment tends to decrease IL-6 plasma levels [23] as well as circulating levels of leptin [25] in PCOS patients, and significantly reduces resistin and TNF-α plasma levels [24] in T2DM patients. Moreover, metformin inhibition of TNF-α-induced IL-6 secretion was abolished after silencing of AMPKα1 in human umbilical vein endothelial cells [26]. Together with our data, this suggests that metformin, via AMPK activation, improves the adipokine profile from human adipocytes in overweight and obese patients. This might contribute to a faster and better improvement of the chronic low grade inflammatory state and therefore insulin resistance in these patients. In turn, adiponectin-induced AMPK activity in skeletal muscles is involved in the regulation of mitochondrial function and oxidative stress, glucose and lipid metabolism, and exercise endurance [27]. The same study further suggests that agonism of adiponectin signaling in muscles provides a new treatment modality for insulin resistance.

In patients with breast cancer, the effects of metformin and AMPK on the adipokine imbalance might diminish tumor progression since the present study showed that adipocyte conditioned medium promoted breast cancer cell proliferation. Decreasing pro-inflammatory adipokine secretion from adipocyte stromal cells will reduce breast cancer cell growth and proliferation. Increasing anti-inflammatory adipokines such as adiponectin will inhibit invasion and migration of breast cancer cells. Indeed, adiponectin targets AMPK activity in breast cancer cells [28]. This leads to an inhibition of the mammalian target of rapamycin (mTOR) pathway and a decrease in protein synthesis, inhibition of adhesion, migration and invasion of breast cancer cells. These results correlate with the activation of AMPK by metformin in breast cancer cells [29]. However and unlike adiponectin, other adipokines (e.g. leptin) might differently alter AMPK activity in various tissues [30]. Therefore, it will be crucial to study the effect of each adipokine on AMPK activity in breast cancer cells.

In conclusion, the present study shows some preliminary but encouraging data to further unravel the specific role of AMPK and adipokines to alleviate obesity-related insulin resistance and breast cancer complications. Activation of AMPK might improve adipokine production triggered by obesity- and diabetes-related factors in human adipocytes. In turn, some adipokines might activate AMPK and increase insulin sensitivity and inhibits breast tumor development.

## Competing interests

The authors declare that they have no competing interests.

## Authors' contributions

JG conceived of the study, participated in its design and coordination, carried out the breast cancer cell proliferation assays and the AMPKalpha1 silencing of the adipocytes, analysed the results and drafted the manuscript. KD carried out the adipocyte and breast cancer cell culture work and the mRNA analyses. DM, UK, BM and MCC participated in the design of the study and coordination, and helped to draft the manuscript. All authors read and approved the final manuscript.

## Acknowledgements and funding

This study was supported by grants from the University of Basel (Forschungsfonds-Förderung für Nachwuchsforschende der Universität Basel) and the Stiftung der Diabetes-Gesellschaft Region Basel to Jean Grisouard, by grants from the Novartis Stiftung für Medizinisch-Biologische Forschung and the Swiss National Research Foundation (PP00P3_123346/1) to Mirjam Christ-Crain and a grant from Sanofi-Aventis to Beat Müller.

We thank Dr. Daniel M. Frey (Department of Surgery, Div. of General Surgery and Surgical Research, University Hospital Basel) and Dr. Ralph Peterli (Department of Surgery, Claraspital Basel) for providing us with human adipose tissue samples.
